# Sarcomatoid variant of urothelial carcinoma in the renal pelvis with brain metastasis: a case report

**DOI:** 10.11604/pamj.2022.41.233.31688

**Published:** 2022-03-22

**Authors:** Hamid Nasrollahi, Faisal Ahmed, Ali Eslahi, Mehrdad Golmoradi Pilehroud, Akbar Safaei, Mohammad Reza Askarpour, AbdolAzim Khorshidi, Soorena Khorshidi

**Affiliations:** 1 Department of Radiation Oncology, School of Medicine, Shiraz University of Medical Sciences, Shiraz, Iran,; 2 Department of Urology, Urology Research Center, Al-Thawra General Hospital, Ibb University of Medical Science, Ibb, Yemen,; 3 Department of Urology, School of Medicine, Shiraz University of Medical Sciences, Shiraz, Iran,; 4 Department of Pathology, School of Medicine, Shiraz University of Medical Sciences, Shiraz, Iran,; 5 Student Research Committee, Shiraz University of Medical Sciences, Shiraz, Iran

**Keywords:** Brain metastasis, chemotherapy, sarcomatoid variant, urothelial carcinoma, case report

## Abstract

Sarcomatoid urothelial carcinoma (UC) of the renal pelvis is rare. It is a high-grade malignant tumor that contains both epithelial and mesenchymal elements. Brain metastases from renal pelvis UC are infrequent and represented in few cases. We report a 68-year-old female with a right renal mass diagnosed as a UC with a sarcomatoid variant. The patient underwent a right radical nephroureterectomy and received chemotherapy. She developed brain metastasis in the left temporal area two months later. Therefore, metastasectomy and palliative brain radiotherapy were performed for her. Sadly, her general condition worsened, and she passed away after one month. Brain metastasis in patients with UC is rare and poorly understood. Therefore, we describe the clinico pathological characteristics, including the clinical follow-up of our case with a focus on the treatment and outcome.

## Introduction

Sarcomatoid urothelial carcinomas (UC) are rare urothelial malignancies with aggressive clinical behaviors, accounting for 0.3% of all urinary tract UC cases [1]. The first case of renal pelvis UC was reported by Piscioli *et al*. in 1984 [2]. Radical surgical resection of the tumor and chemotherapy remains the optimal treatment for this tumor. Brain metastasis is extremely rare in UC patients and represents 0.4% to 0.6% of all urinary tract UC cases [3]. To our knowledge, just a few cases of sarcomatoid UC of the renal pelvis with brain metastasis have been reported [4]. Therefore, we describe the clinico pathological characteristics, including the clinical follow-up of our case with a focus on the treatment and outcome.

## Patient and observation

**Patient information:** a 68-year-old woman presented with mild right-side flank pain and intermittent hematuria in the last month. Her pain was not colicky and did not interfere with her routine life. There were no other urologic problems, such as dysuria, frequency, or dribbling. Her medical history was not significant.

**Clinical findings:** the patient vital signs were stable (blood pressure: 120/70mmHg, respiratory rate: 14 respirations per minute, pulse rate: 61 beats per minute). She was pale and had mild right flank tenderness.

**Figure 1:**
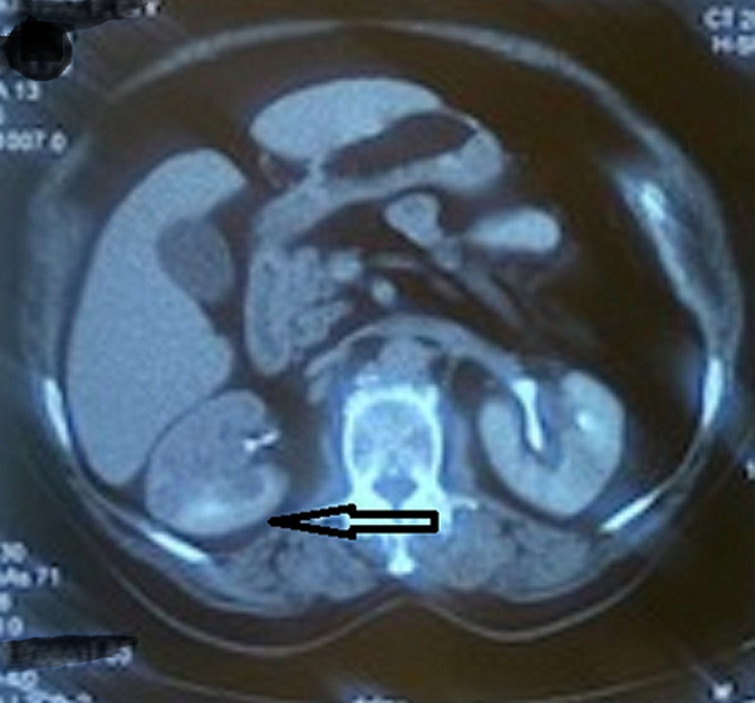
abdominal computed tomography (CT) scan demonstrates a mass in the right renal pelvis (arrow)

**Diagnostic assessment:** urine analysis showed microscopic hematuria, and other blood tests and urine culture were normal. According to her signs and symptoms, an ultrasound was requested that showed a 5x4cm hypoechoic mass in the right mid-pole and lower pole of the kidney. Chest and abdominal computed tomography (CT) scans were done, showing a 5×4cm enhanced irregular soft tissue mass in the mid-pole extended to the lower pole of the right kidney without evidence of metastases (Figure 1).

**Figure 2:**
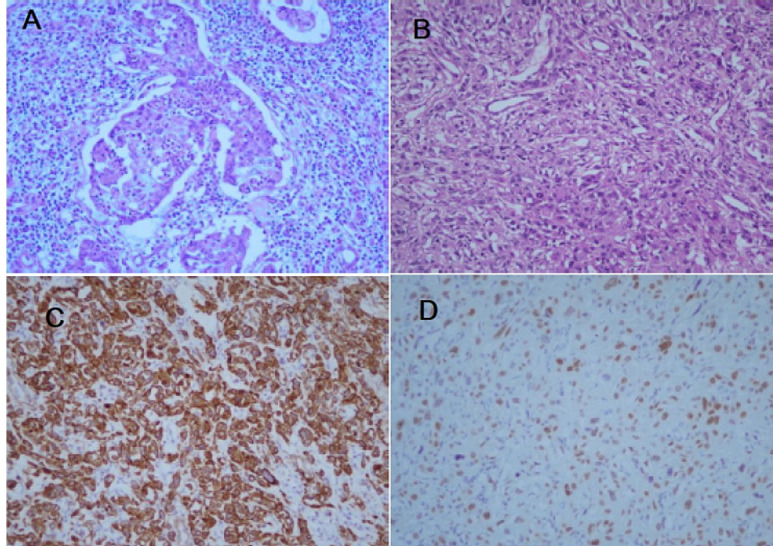
sections of renal mass show infiltrative epithelial nests in renal parenchyma: A) with foci of sarcomatoid feature; B) immunohistochemistry shows a positive reaction for pan-cytokeratin; C) GATA3; D) X200

**Therapeutic interventions:** she underwent a right radical nephroureterectomy. The pathological report confirmed the diagnosis of high-grade UC with sarcomatoid differentiation. An immuno histochemistry (IHC) study was done, revealing a positive reaction for pan-cytokeratin and GATA 3 on both epithelial and sarcomatoid components and a negative reaction for CK5/6, P63, PAX8, and CD10 confirming the diagnosis of the sarcomatoid variant of UC (Figure 2).

**Figure 3:**
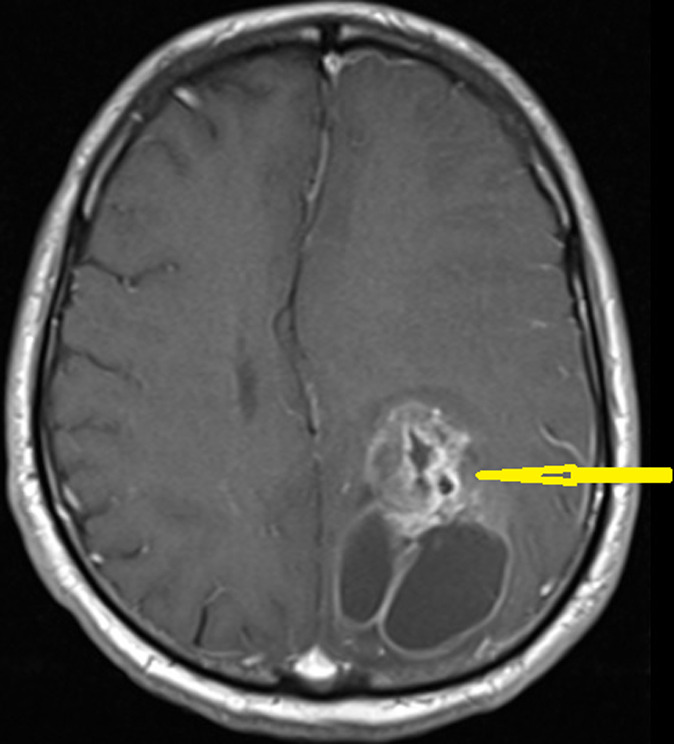
brain magnetic resonance imaging (MRI) shows brain metastasis (arrow)

**Follow-up and outcome of the interventions:** the patient was referred to the oncology clinic for adjuvant treatment. She received chemotherapy with gemcitabine (1000mg/m^2^ day 1 and 8) and cisplatin (75mg/m^2^ day 1); she developed neutropenic after the 2^nd^ and 3^rd^ cycles of chemotherapy and was unable to continue the chemotherapy. Thus, tumor bed radiotherapy was started for her. She received 44 grays (Gy) in 22 fractions to the tumor bed and regional lymph nodes. After two months, she developed severe headaches, and magnetic resonance imaging (MRI) showed brain metastasis (in the left temporal lobe, 9mm peripheral ring-enhancing lesion with crescent increased attenuation and adjacent edema) (Figure 3). Then a metastasectomy was performed, and the pathology report showed metastatic carcinoma of the UC. The patient received palliative brain radiotherapy with a dose of 30 grays (Gy) in ten fractions, but her general condition worsened, and she passed away after one month.

**Patient perspective:** the patient's son mentioned that “I would like to thank everyone from the nurses to the physicians that helped us take care of my mother, from the day she went to the hospital to the day that she left us”.

**Informed consent:** a written informed consent was obtained from the patient's family for participation in our study.

## Discussion

Clinically, primary renal pelvis sarcomatoid UC is rare. The average age of presentation of this disease is over 50 years, with a male-to-female ratio of approximately 2/3. Symptoms of this tumor such as flank pain, gross hematuria, hydronephrosis, and abdominal mass are frequently like those of other renal tumors. However, the higher-grade and stage, metastases at presentation, poor prognosis as well as aggressive nature are the main characteristics of sarcomatoid UC [4]. Our patient was a 68-year-old woman who had right flank pain and hematuria when she came in. The sarcomatoid variant of UC is rare and aggressive, with approximately 100 reported cases, the vast majority of which occur in the urinary bladder. These tumors are also known as carcinosarcoma and spindle cell carcinoma [5]. According to Lopez-Beltran *et al*. microscopically sarcomatoid urothelial carcinoma has a urothelial, glandular, or small cell component with varying degrees of differentiation, and carcinoma in situ is found in 30% of these cases [6]. Similarly, in our case, epithelial and mesenchymal elements were found. There has been no agreement between published papers on UC treatment with sarcomatoid variants. However, the main suggested treatment with prolonged patient survival is radical surgery with systemic chemotherapy and radiation therapy [7]. A recent study found that upper urinary tract sarcomatoid carcinomas expressed the epidermal growth factor receptor (EGFR), implying that molecular targeted therapy may be a promising potential therapeutic route [8]. The regimen suggested for metastatic sarcomatoid UC is gemcitabine and doxorubicin with an objective response rate of 16% and a median progression-free survival of 3.5 months [9]. In our case, the radical nephroureterectomy was performed, followed by chemotherapy administration. However, the patient developed a neutropenic fever. For that, the patient was switched to tumor bed radiotherapy.

Brain metastasis is extremely rare in UC patients and represents 0.4% to 0.6% of all urinary tract UC cases. Furthermore, the frontal lobe, parietal lobe, temporal lobe, basal ganglia or thalamus, occipital lobe, brainstem, and cerebellum were the most common sites for brain metastases, respectively [3]. Headache is the most common symptom in patients with brain metastasis, affecting roughly half of all patients. Additionally, focal neurologic dysfunction may represent 20% to 40% of patients with the most common hemiparesis [10]. Brain MRI is the best radiologic modality for detecting brain metastases because brain CT may underestimate the number of metastatic brain lesions [11]. The favorable prognostic factors for brain metastasis are younger age, high preoperative Karnofsky performance scale score, maximal radio-surgical dose to tumor margin, less sarcomatoid components, graded prognostic assessment (GPA) score of less than 2, and the long interval between the diagnosis of renal cancer and the onset of brain metastasis [1,3]. Previously published articles estimate that brain metastasis has a lower prognosis than other metastasis sites, with an average survival rate of 4-5 months following diagnosis and treatment of brain lesions [12], just like our patient who passed away after one month. The best cure for brain metastasis is still unknown. Surgical resection, whole-brain radiation therapy, and stereotactic radiosurgery are the most popular procedures [13]. Thanks to fractionated radiation therapy, the median period of life has increased from 3 to 6 months. The majority of radiation oncology centers administer 30 to 40 gray (Gy) doses in 10 to 20 fractions [3]. In our patients, radiotherapy following metastasectomy was an option. Nobuyuki *et al*. presented a sarcomatoid variant UC in a 66-year-old man with brain metastasis who passed away after 4.8 months of treatment [1]. On the other hand, Fang *et al*. reported a left kidney UC with brain metastasis in a 55-year-old woman. The patient developed left frontal lobe brain metastasis ten months after renal surgery. For that, metastasectomy was performed, and the patient had no sequelae within one year of follow-up [14].

## Conclusion

Despite the rarity of UC metastasis to the brain, it is essential to consider the urinary tract system as the primary site of brain metastasis. Additionally, sarcomatoid UC with brain metastasis has a poor prognosis and low patient survival.
